# The trap bag concept of afterglow luminescence

**DOI:** 10.1038/s41598-017-07789-2

**Published:** 2017-08-16

**Authors:** Yih-Ping Huang, Jeng-Wen Lin

**Affiliations:** Department of Civil Engineering, Feng Chia University, 100, Wenhwa Rd., Taichung, 407 Taiwan

## Abstract

This article explains the behavior of afterglow luminescence using the trap bag concept, in which a constant phosphor dose contains a presumed bag with the ability to capture or release electrons through its opening. Luminescence is emitted as the bag releases the captured electrons. The electron-holding capacity is determined by the irradiation conditions, the width of the opening, and the electron activation; these factors are inherent properties of the long persistent luminescence (PLUM) dose and are affected by the thermal status. During the afterglow stage, higher temperatures may result in a wider opening and increased activation of electrons released from the bag, thus creating a higher light intensity and leading to the quicker exhaustion of the electrons within. In contrast, the opposite phenomenon will occur at lower temperatures. This article provides a detailed explanation of the trap bag concept at various thermal statuses and provides a method for delaying the afterglow peak profile through temperature change. Experimental tests were performed to confirm the proposed concept.

## Introduction

Strontium aluminate phosphors (SrAl_2_O_4_: Eu^2+^, Dy^3+^) demonstrate excellent afterglow luminescence properties^1^. These include an increased luminescence duration, high light intensity, non-toxicity, and good chemical resistance. Han *et al*.^2^ describes the luminescence characteristics as electron carriers trap and thermally release electrons at room temperature. With proper excitation, electrons (or photons) and traps are produced in pairs; after removing the incident energy, some captured electrons are thermally released in order to reach a state of equilibrium with gradual emission of light. Meléndrez *et al*.^3^ suggests that irradiation temperature is the key factor affecting the time-integrated intensity of long persistent luminescence (PLUM) emission in strontium aluminate phosphors. Kumar *et al*.^4^ found that during afterglow, a higher heating rate increases the peak value of the glow curve and increases the speed at which material luminescence glows. With a constant phosphor dose, and under changing temperature and irradiation conditions, most studies agree that temperature is the dominant factor affecting afterglow behavior. When using afterglow materials for stress measurement, the term mechanoluminescence (ML) is used in many studies to emphasize the differences between stress-induced and thermal-induced luminescence behavior. For example, Jia *et al*.^5^ found that while dynamic stress can induce ML, static stress cannot. However, Chandra *et al*.^6^ showed that it was impossible to dislocate the nanoparticles of the phosphor doses, so the appearance of ML may not be attributed to mechanical or electrostatic interactions.

In previous afterglow behavior studies, the level positions of the trapped electrons have been exploited to predict some of the basic behaviors of a phosphor dose. However, the electron level positions alone do not appear to be enough to provide a full explanation for all electron storage and luminescence problems^7^. Studies related to electron level positions in the afterglow are the most popular subject of investigations in the phosphor material field. In contrast, this article provides a simple and novel concept of a bag with an opening to explain the afterglow decay profile that correlates with temperature change, while exploiting applications such as stress measurement in order to produce a postponed afterglow decay curve. A simple monotonic relationship with an increase in temperature for the glow curves only exists over a limited temperature range. In other words, there is no guarantee that a high temperature will lead to a high curve location on the profile plot. During the afterglow stage, a temperature change can alter the curve location and its profile. This phenomenon is important in stress measurement as well as in other applications.

## Concept

To describe afterglow luminescence behavior, this article proposes the trap bag concept: for every phosphor dose there is a presumed bag with an opening with some number of captured electrons (also called photons) inside. Gradually, these electrons are thermally released through the opening to emit luminescence until they are exhausted or reach some equilibrium state. The capability of the bag is one of the inherent characteristics of an afterglow dose. The size of the bag’s opening and its ability to activate electrons are decided by the thermal status. During the afterglow stage, higher temperatures lead to a wider opening and more electrons are activated and released from the bag to create more active luminescence. The following three statements provide the main framework for this concept:

1. There is a trap bag in every individual phosphor dose.
2. Each trap bag has an opening and its size determines the number of electrons (photons) to pass through. The width of the opening is affected by temperature; higher temperature creates a wider opening, and vice versa.
3. During the charging process, activated electrons can jump into the trap bag for that dose until it is full or reaches equilibrium status. In the afterglow stage, these electrons can be thermally released and emit luminescence until they are exhausted or reach equilibrium. Equilibrium status in a trap bag means that in a light environment, the charging and afterglow processes occur at the same time and the number of input and output electrons is balanced. The activity of those electrons is affected by temperature; that is to say, electrons pass more easily through the bag’s opening at a higher temperature. On the other hand, decreasing the temperature can result in a tightening of the bag’s opening and a reduction of electron activation that cuts down luminescent intensity and minimizes the electron loss rate.

The three statements above can be used to explain the following three luminescent phenomena:

1. In the charging process, the source type, intensity, and temperature can affect the quantity of captured electrons and the size of the bag’s opening. At higher temperatures, it can spur the activation of electrons significantly and widen the bag’s opening, which results in the capture of more electrons into the bag. However, if the opening is too wide, the captured electrons may simultaneously escape, thus reducing the bag’s containment capability. In other words, there is no guarantee that a higher temperature will lead to improved afterglow behavior, such as a higher luminescent intensity or a longer luminescence duration.
2. After removing the charging light source, electrons captured inside the bag can be released through the opening to emit luminescence. Thermality is the dominant factor in the process; it determines the number of electrons released and the wideness of the opening.
3. By carefully controlling temperature, it is possible to hold those captured electrons in the bag for some time and to release them later on. In other words, after terminating the excitation process, the afterglow behavior can be frozen by lowering the temperature and resumed later by increasing the temperature.

## Experimental tests

### Sample preparation

Most studies have focused on powder samples; however, thin film phosphors have several advantages over them, such as an easier testing process, a direct relationship with marketplace products, better thermal stability, and better adhesion to film surfaces^8^. This study used green emitting strontium aluminate phosphors (SrAl_2_O_4_: Eu^2+^, Dy^3+^) to produce 0.5 mm thickness thin films for experimental tests. The phosphors are commercially available for purchase in the powder form. The phosphor particle with a diameter of 0.1 mm was used after mixing with 50% of PET resin, i.e., the ratio of PET resin versus phosphors mixture is 1:1 in weight. To create the patches, a mesh print technique is introduced through printing layer by layer on top of a substrate, with each layer being approximately 0.1-mm thick.

Before commencing the charging process, the samples were preheated at 100 °C in the dark for 5 h in order to empty the traps and erase any residual or acquired luminescence^9^. The temperature range was set between −10 °C to 110 °C in the following measurement tests. A standard D65 light source with 3000 LUX on the target was provided in order to irradiate the samples for 5 min. DIN 67510^10^ luminescence standards were adopted except the irradiation intensity was increased up to 3000 lx, and the temperature was varied as required. To change temperature quickly for a sample, two ovens were used. Inside the oven were four 6500-K 100-W intensity-adjustable Xenon lamps that were attached to the top and sidewalls to excite afterglow specimens. The lamps could be switched on independently to meet the required luminescence intensity. For each oven, a computer-controlled luminance meters (Konica Minolta LS-100) was used to measure the luminous intensity of the afterglow specimens^11^. Then, the afterglow decay curves were recorded under constant or varying temperatures.

### Curve modeling

The amount of measured data from an afterglow test can be large. For example, this study reads data from a luminance meter every three seconds. Multiple single exponential equations are commonly used to fit the data^11, 12^, as demonstrated in equation (1), which includes the offset term (*I*_0_), indicating that it is unable to block the ambient light entirely^13–15^.

1 $$I={I}_{0}+\sum _{i=1}^{n}{\alpha }_{i}\,\exp (\frac{-t}{{\tau }_{i}})$$

in which *I* is the light intensity at time, *t*, after switching off the excitation; illumination, *I*_*0*_, is the offset term representing the residual light; *α*_*i*_ is a constant; and *τ*_*i*_ is a decay constant. If the data is obtained from an ideal completely dark room, *I*_*0*_ should be zero, and equation (2) is obtained:

2 $$I=\sum _{i=1}^{n}{\alpha }_{i}\,\exp (\frac{-t}{{\tau }_{i}})$$

Using different n values results in the following three equations as equations (3, 4 and 5).

3 $$I={I}_{0}+{\alpha }_{1}\,\exp (-t/{\tau }_{1})$$

4 $$I={I}_{0}+{\alpha }_{1}\,\exp (-t/{\tau }_{1})+{\alpha }_{2}\,\exp (-t/{\tau }_{2})$$

5 $$I={I}_{0}+{\alpha }_{1}\,\exp (-t/{\tau }_{1})+{\alpha }_{2}\,\exp (-t/{\tau }_{2})+{\alpha }_{3}\,\exp (-t/{\tau }_{3})$$

It is, however, up to the user to adopt appropriate multiple single exponential equations, as shown in the above three equations, for the associated numerical simulation for each case. Furthermore, whether to consider an *I*_*0*_ term should be decided by the user. Detailed discussions on numerical simulation and physical interpolation related to this subject can be found in other manuscripts^11–15^. Equation (5) without an *I*_*0*_ term is used throughout this article for the modeling of afterglow curves. Logarithmic scale can be beneficial for a single-exponential equation. However, no obvious advantage could be found by using logarithmic scale to treat a set of data modeled with multiple-exponential equations.

### Test results

In this study, the effect of irradiation temperature on PLUM behavior was investigated to assess its capability to alter the luminescent response. In the experimental tests, the afterglow decay profiles of the samples were measured at both constant and varying temperatures as described below:

1. Various constant temperatures were used throughout the tests. These included room temperature and several other temperatures ranging from −10 °C to 110 °C.
2. Varying temperatures were used throughout a single afterglow test.

**Figure 1:**
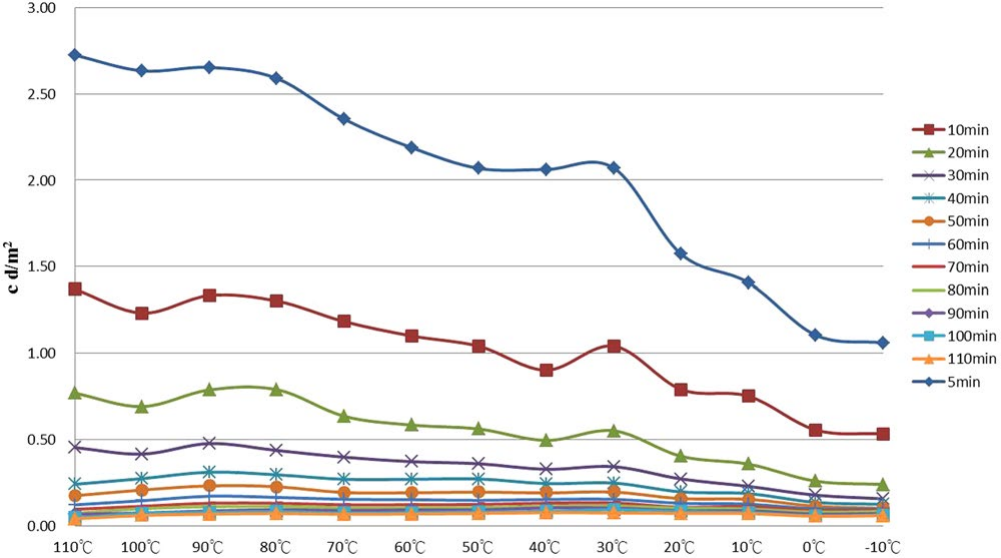
A TL-temperature plot for tests at a range of constant temperatures. The right hand side shows symbols for various durations, and the bottom scale indicates the constant temperatures used throughout the tests.

**Figure 2:**
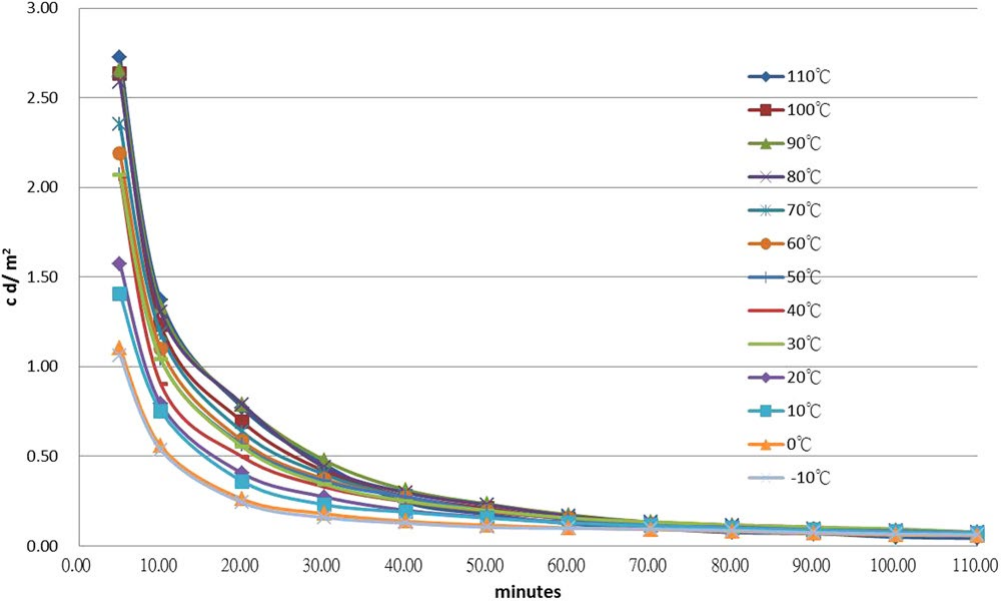
The TL-time plot of Fig. 1. The right hand side shows symbols for various temperatures, the bottom scale indicates the duration of the afterglow profile.

Figure 1 shows the thermoluminescence (TL)-temperature plot for each test under various constant temperatures. The corresponding TL-time plot is shown in Fig. 2. In these two figures, the afterglow intensity variation decreases with decreasing temperature between −10 °C and 110 °C, with maximum intensity at approximately 90 °C, and a second intensity peak at approximately 30 °C. Similar test results were obtained by Xiao *et al*.^16^ and Hölsä *et al*.^7^ who described the behavior as a monotonic increase in intensity with increasing temperature, which peaked at approximately 110 °C, and then decreased as the temperature was further increased. However, a report by Meléndrez *et al*.^3^ was inconsistent with the above findings, stating that the PLUM in SrAl_2_O_4_: Eu^2+^, Dy^3+^ exhibited a monotonic decrease as temperatures rose above room temperature. These contrasting results may be due to the light source, intensity, the duration of exposure, or phosphor dose type. The afterglow curve of a constant dose is influenced by the input of the pump light; it cannot be well characterized without consideration of its charging processes and efficiency, which includes the light source, intensity, and degree of saturation^9^.

**Figure 3:**
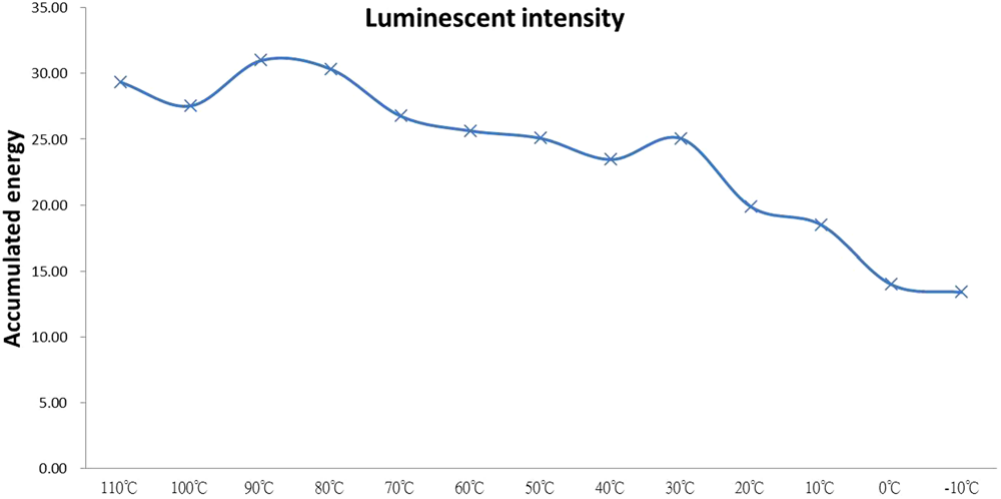
The TL-time-integrated intensity of Fig. 1. There are two peaks visible in the curve.

Another plot form of Figs 1 and 2 is illustrated in Fig. 3, demonstrating that temperature is a key factor affecting the time-integrated intensity of the afterglow in a sample with varying constant temperatures. The integrated intensity increases monotonically at approximately 30 °C, and then drops slightly before peaking at approximately 90 °C. Under constant dose and irradiation conditions, this figure shows the relationship between the temperature and the captured electron number (or photon energy) in a trap bag. In order to obtain the best afterglow profile for a constant temperature, 90 °C is the best option for the thin film used in this article. Within the temperature range investigated, 90 °C provides the best luminescence conditions for the bag’s opening as well as electron activation levels when applying the proposed trap bag concept. Even though temperatures higher than 90 °C can still enhance the electron activation level, they can also reduce the electron holding capability of a trap bag by inducing a wider opening. This explanation is also valid at 30 °C. In the low temperature range between 0 °C and −10 °C, after 30 min into the afterglow process the luminescence intensity becomes very low. This phenomenon indicates that low temperature can result in a tightening of the bag’s opening and a reduction of electron activation, thus minimizing the number of electrons released from the bag. This implies that electrons can be held in the bag by inducing low temperatures and then released with high temperatures. The following experimental tests will confirm this concept.

**Figure 4:**
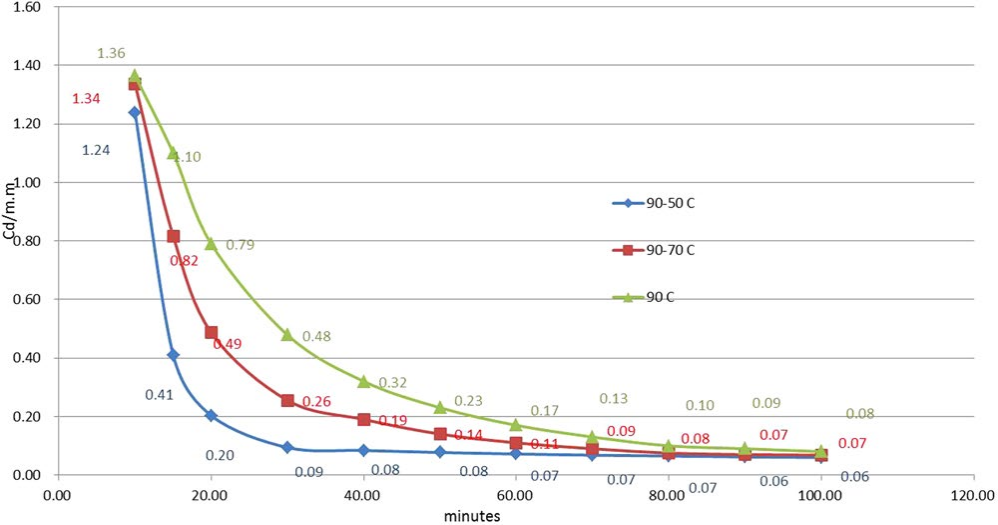
The TL-time plot for tests performed with a temperature decrease during the process. The top curve is the original with a constant 90 °C throughout its lifetime. The temperatures for the middle and bottom curves were decreased to 70 °C and 50 °C respectively 10 minutes after excitation was terminated.

**Figure 5:**
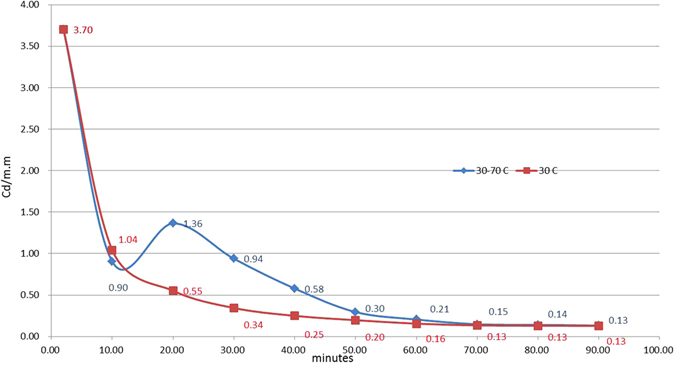
TL-time plot for tests performed with a temperature increase during afterglow. The square symbol curve is the afterglow curve for a film at a constant 30 °C throughout the lifetime. For the diamond symbol curve, the temperature was increased to 70 °C 10 minutes after excitation was terminated.

**Figure 6:**
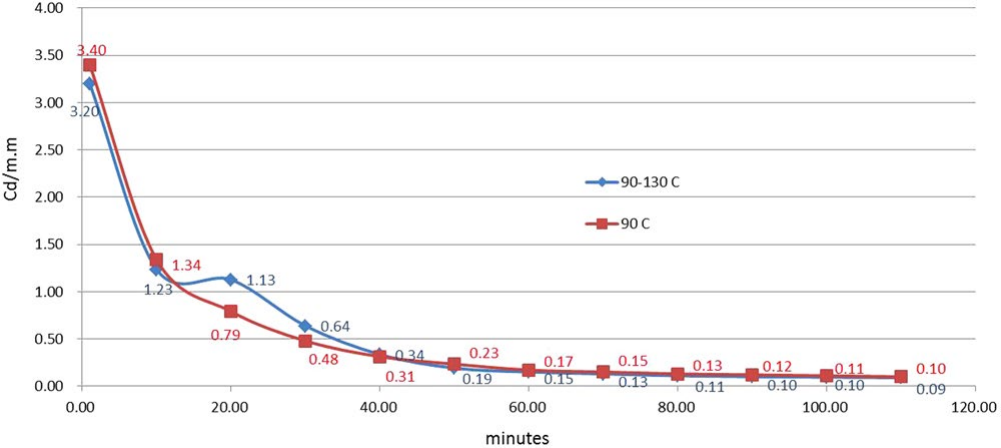
TL-time plot for tests performed with a temperature increase during afterglow. The square symbol curve is the afterglow curve for a film at a constant 90 °C throughout the lifetime. For the diamond symbol curve, the temperature was increased to 130 °C 10 minutes after excitation was terminated.

After excitation, captured electrons are prepared for the emitting source. Using different temperatures during the emitting process can cause variance in the afterglow decay curves. Kumar *et al*.^4^ demonstrated that the area under the curve in a TL-time plot is conserved. However, Fig. 4 shows results that are inconsistent with the findings of this study. The figure shows a TL-time plot with three decay curves from tests with initial irradiation at 90 °C. 10 min into the afterglow, the irradiation temperature for two of the tests was lowered to 70 °C and 50 °C, respectively. The area in the constant 90 °C case is the largest among the three curves, followed by 70 °C and 50 °C. Lowering the temperature during the afterglow process can cut down the associated intensity-time integrated area. On the other hand, Figs 5 and 6 show the results of varying tests that commenced at between 30 °C and 90 °C respectively with an increase of 40 °C during the afterglow process. An increase in intensity-time integration areas is found in both figures. Figures 4 and 5 show that the area under the glow curve in a TL-time plot is not conserved, it increases with rising temperature and vice versa.

**Figure 7:**
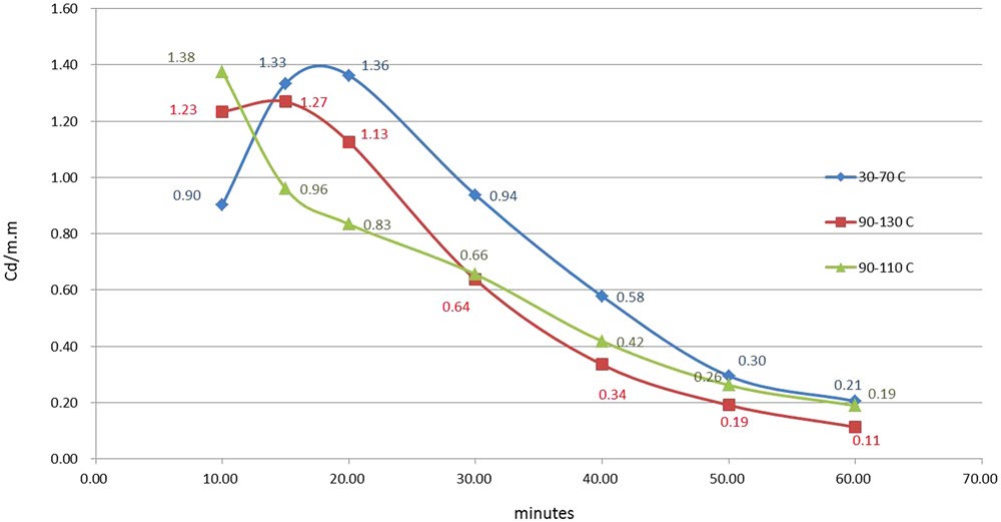
TL-time plot showing a decrease in luminescent intensity with an increase in temperature. The two cross curves are a part of Figs 5 and 6 respectively. The triangle symbol curve is from a test that began at 90 °C with a temperature increase to 110 °C 10 minutes later.

The two cross sign curves in Fig. 7 are parts of Figs 5 and 6, respectively. The triangle sign curve is from a test that began at 90 °C with a temperature increase to 110 °C 10 min into the afterglow. This triangle symbol curve shows that an increase in temperature can result in a decrease in luminescent intensity and its behavior agrees with Fig. 3. Again, this figure indicates that there is no monotonic relationship between the temperature and the luminescent intensity. The test results shown in Figs 4, 5, 6 and 7 are inconsistent with the above statements that the area under the TL-time curve is conserved, as proposed by Kumar *et al*.^4^. One obvious problem: where do those surplus or deficit electrons (or energy) come from and where are they heading?

**Figure 8:**
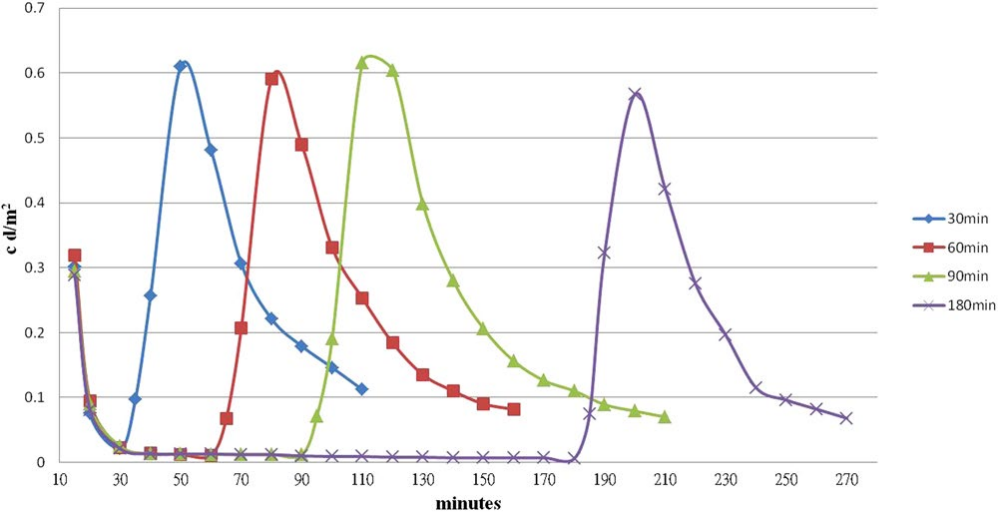
TL-time plot showing four curves that represent tests performed with an initial excitement at 90 °C, then 10 minutes later after ending excitation their temperatures were lowered to 0 °C for 30, 60, 90, and 180 minutes respectively before increasing the temperature back to 90 °C.

Before answering the above questions, an interesting result is noted in Fig. 8. Decay curves for a thin film can be seen, initially excited at 90 °C and decreased to 0 °C 10 min later, then resumed at 90 °C 30, 60, 90, and 180 min later, respectively. In those four cases, the intensity-time integration areas were almost the same. In other words, the total numbers of captured electrons (or photon energy) were equivalent for each test, and the electron consumption during the low-temperature period was minimal. It seems that temperatures below 0 °C can tighten the opening of a trap bag and almost completely contain the held electrons, and that the bag can be opened in order to resume afterglow behavior by increasing the temperature.

**Figure 9:**
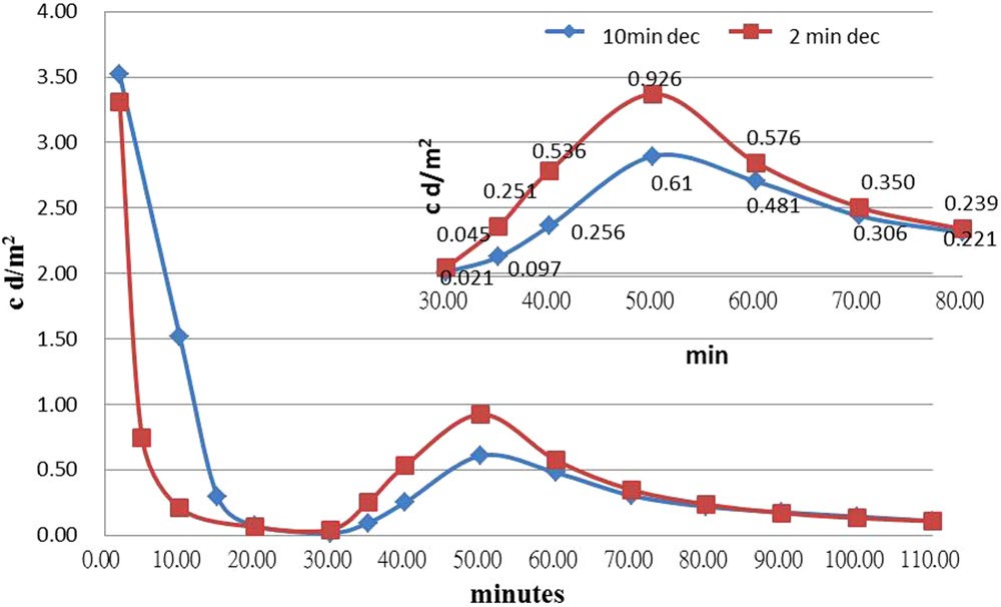
A TL-time plot with two curves with an initial irradiation at 90 °C, which was then decreased to 0 °C after at 2 and 10 minutes for the rectangle and diamond symbol cases respectively.

Figure 9 shows a sample of two decay curves from tests in which the temperature was dropped to 0 °C 2 and 10 min subsequent to the afterglow process. After 30 min, the temperature was increased to the original temperature of 90 °C. This shows that the earlier the temperature drops, the more electrons that can be retained in the trap bag. This figure indicates that minimizing the electron consumption at the early emission stage can be beneficial to the afterglow as the temperature rises.

## Discussion and Conclusion

The exact luminescent model of the popularly accepted shallow and deep traps concept is still far from being well confirmed^2^, and the details of trapping during the charging process still need to be better understood^9^. A novel and rational concept is proposed in this article as the trap bag concept describes the afterglow behavior of phosphorous material, in order to perform related analysis as a function of temperature that may explain the associated behaviors.

The irradiation results can be optimized through appropriate choice of temperatures, although the authors doubt that this temperature is not unitary under different situations such as low input light intensity, varying light sources, and cases of extreme temperature. Once the electrons are captured in the trap bag, they are the only source of emitted light while escaping gradually from the bag’s opening.

In the afterglow stage, if no input light is allowed, two factors influence the emitting rate: the wideness of the opening and the activation of the electrons - both are determined by temperature. In general, higher temperatures can cause wider openings, stronger activation levels, and higher luminescent intensity.

The afterglow of a phosphor product acts differently for indoor and outdoor usage due to the temperature difference. In terms of stress-related measurements, some appropriate temperatures can be used at the irradiation stage to capture as many electrons as possible. When terminating the input light, one can immediately lower the temperature to 0 °C to freeze the afterglow behavior and minimize energy consumption. This frozen status can be maintained for several hours and afterglow behavior will resume as the temperature rises.
